# Supplementary material for: Interventions to Increase Physical Activity in Community-Dwelling Older Adults in Regional and Rural Areas: A Realist Synthesis Review Protocol
Source: Methods Protoc. 2023 Mar 12;6(2):29. doi: 10.3390/mps6020029 (PMC10037574; doi:10.3390/mps6020029)
Supplement: Supplementary file 1 [file mps-06-00029-s001.zip › mps-2226321-supplementary.pdf]

Data Extraction

The aim of this form is to extract data from the identified studies, to develop one or more explanatory theories to account for Context-Mechanism-Outcome Configurations (CMOc) and develop an understanding of how our CMOCs fit with within the framework of increasing physical activity in older adults.

In realist approaches the accumulation of knowledge is around our understanding of the behaviour of mechanisms in different contexts and how and why this interaction of context and mechanism causes different outcomes.[1]

Please complete the details below, for the study you will be reviewing.

Reviewer Name	
Reference	
Setting	
Recruitment	
Participant Mean Age (±SD)	
Study Design	
Intervention Name (if applicable) and Details	
Time Frame and Follow-up	
Physical Activity Outcome(s)	
Other Outcomes	

Process

In the following 3 tables, identify the Context, Mechanism and Outcomes (CMO) of the intervention in the study you have reviewed. Definitions of CMO and examples are supplied in the tables.

Table 1 - Context

Including (but not limited to) beliefs, social and cultural norms, regulations and economic factors.[2]<br>For example[3]:<br>▪ Individual capabilities (i.e. values, roles, knowledge, purpose).<br>▪ Interpersonal relationships supporting the interventions (i.e. communication, collaboration, network, influences).<br>▪ Institutional settings (i.e. informal rules, organisational culture, leadership, resource allocation, local priorities).<br>▪ Infra-structural system (i.e. political support).<br>▪ Country and rurality (i.e. small or large/rural vs regional vs remote).<br>▪ Funding context or source (i.e. free, personalised, group vs. individual, government funded).

Table 2 – Mechanisms

"An element of reasoning and/or reactions of an individual or collective agents(s) in regard of resources available in a given context to bring about changes through the implementation of an intervention"[4]<br>Check what are the mediators to produce outcomes (not only primary, but also secondary or unintended outcomes).

Table 3 – Outcome

Please make note of all (intended or not) outcomes (primary, secondary, etc) (i.e. increase self-efficacy or confidence in ability to be physically active; increased skills or confidence using technology; increase in socialisation, social support structures and social connectiveness; increase in perceived physical health; increase in total physical activity participation in minutes/distance/frequency etc; change in time spent in different physical activity intensities; increased ability to establish behavioural change goals; increase in readiness-to-change behaviour classification).<br><br>Please also classify physical activity outcomes as the following:<br>1. Increase in physical activity participation.<br>2. No change in physical activity participation.<br>3. Decrease in physical activity participation.

Table 4 – Context-Mechanism-Outcome Configurations (CMOc)

Please detail the CMOc (partial or complete) for the study you reviewed and your rationale/explanation?<br>C + M = O (i.e. an aqua aerobics class **[context]** + council funded free class offer for seniors **[mechanism]** equals an increase in uptake of physical activity **[outcome]**).<br>C → M → O. Context triggers Mechanism (i.e. Medicare funded 5 allied health visits under chronic disease care plan **[context]** triggers an increase in exercise physiologist consultation/individualised exercise plan **[mechanism]** which triggers an increase in physical activity **[outcome]**).<br>Differentiate trigger (→) from plus (+) which is more like process. Please also note that sometimes C can be an M or an O or vice versa or both.

Table 5 – Assessment of Study Rigour

Is the evidence provided in this paper sufficiently trustworthy and rigorous enough to be included in the realist synthesis? Consider issues of sample size, data collection, data analysis and claims made? **YES / NO** (comments below)

References

1. Wong G, Westhorp G, Manzano A, Greenhalgh J, Jagosh J, Greenhalgh T. (2016). RAMESES II reporting standards for realist evaluations. *BMC Medicine*, **14(1)**, 1-18.
2. Wong G, Greenhalgh T, Westhorp G, Pawson R. (2012). Realist methods in medical education research: what are they and what can they contribute? *Medical Education*, **46(1)**, 89-96.
3. Macfarlane F, Greenhalgh T, Humphrey C, Hughes J, Butler C, Pawson R. (2011). A new workforce in the making?: A case study of strategic human resource management in a whole-system change effort in healthcare. *Journal of Health Organization and Management*, **25(1)**, 55-72.
4. Lacouture A, Breton E, Guichard A, Ridde V. (2015). The concept of mechanism from a realist approach: a scoping review to facilitate its operationalization in public health program evaluation. *Implementation Science*, **10(1)**, 1-10.
